# Geographic dimensions of a health network dedicated to occupational and work related diseases

**DOI:** 10.1186/s12942-016-0063-7

**Published:** 2016-09-27

**Authors:** Marie Delaunay, Vincent Godard, Mélina Le Barbier, Annabelle Gilg Soit Ilg, Cédric Aubert, Anne Maître, Damien Barbeau, Vincent Bonneterre

**Affiliations:** 1 TIMC Research Laboratory (UMR CNRS 5525), EPSP Team (Environnement et Prédiction de la Santé des Populations), Université Grenoble Alpes, 38041 Grenoble, France; 2 LADYSS Research Laboratory (UMR CNRS 7533) (Laboratoire Dynamiques sociales et recomposition des espaces), Université Paris 8, 93526 Saint-Denis, France; 3 MSH Paris Nord (Maison des Sciences de l’Homme), Universités Paris 8 et Paris 13, 93210 Saint-Denis, France; 4 Mission RNV3P (French Network for Occupational Diseases Prevention and Vigilance Network), ANSES (French Agency for Health Safety in Food, Environment and Work), 94701 Maisons-Alfort Cedex, France; 5 Occupational Health Department, Santé Publique France (French National Public Health Agency), 94415 Saint-Maurice Cedex, France; 6 Occupational Health Department, CHU Grenoble-Alpes (Grenoble Teaching Hospital), 38043 Grenoble, France; 7 Occupational and Environmental Toxicology Laboratory, CHU Grenoble-Alpes (Grenoble Teaching Hospital), 38043 Grenoble, France

**Keywords:** Occupational health, Occupational diseases, Surveillance network, Stakeholders, Geographic Information System, Spatial analysis, France

## Abstract

**Background:**

Although introduced nearly 40 years ago, Geographic Information Systems (GISs) have never been used to study Occupational Health information regarding the different types, scale or sources of data. The geographic distribution of occupational diseases and underlying work activities were always analyzed independently. Our aim was to consider the French Network of Occupational Disease (OD) clinics, namely the “French National OD Surveillance and Prevention Network” (rnv3p) as a spatial object in order to describe its catchment.

**Methods:**

We mapped rnv3p observations at the workplace level. We initially analyzed rnv3p capture with reference to its own data, then to the underlying workforce (INSEE “Employment Areas”), and finally compared its capture of one emblematic occupational disease (mesothelioma) to an external dataset provided by a surveillance system thought to be exhaustive (PNSM).

**Results:**

While the whole country is covered by the network, the density of observations decreases with increase in the distance from the 31 OD clinics (located within the main French cities). Taking into account the underlying workforce, we show that the probability to capture and investigation of OD (assessed by rates of OD per 10,000 workers) also presents large discrepancies between OD clinics. This capture rate might also show differences according to the disease, as exemplified by mesothelioma.

**Conclusion:**

The geographic approach to this network, enhanced by the possibilities provided by the GIS tool, allow a better understanding of the coverage of this network at a national level, as well as the visualization of capture rates for all OD clinics. Highlighting geographic and thematic shading zones bring new perspectives to the analysis of occupational health data, and should improve occupational health vigilance and surveillance.

**Electronic supplementary material:**

The online version of this article (doi:10.1186/s12942-016-0063-7) contains supplementary material, which is available to authorized users.

## Background

The Occupational Health (OH) field is complex because it combines many different types of data (activity sector, occupations, risk exposures, diseases), available at different levels (municipalities, activity territories, employment areas, regions, etc.) and from different partners (insurers, stakeholders, monitoring systems). These multiple sources of data, formalized or not, have always been analysed independently, ignoring in particular the associated geographic dimension (zones of activity).

Even though OH is spatially determined, linked to the location of particular sectors of activity, only a few papers have addressed it. Although the use of the Geographic Information System (GIS) was discussed in studies describing the spatial distribution of some activity sectors and work related problems [1–4], very few papers have followed [5].

Very recently a “proof of concept” paper [6] showed that the use of GIS was a useful way to integrate, analyse and present OH data, from a national to a local level (macro approach), but also within a single workplace (micro approach). This article discussed how these methods and the maps derived from them could be useful for clinicians, epidemiologists, prevention stakeholders, and surveillance authorities. For a better assessment of the importance of occupational diseases within a specific territory, it is necessary to take into account the underlying active worker population (“denominator”) and its distribution according to activity sectors. As an example the authors started to map the active population in one specific sector of activity of concern (workplaces and the number of salaried workers in each). To give perspective, further steps identified were to generalize this approach to the whole active population, and to use denominator figures to calculate and map complementary information such as the expected number of occupational diseases, and indicators comparing observed cases and expected number of cases. When a data source is not expected to be exhaustive in its capture (preventing the calculation of incidence rates) such indicators could still be very useful to: (1) describe the catchment area of health providers specializing in the diagnosis and care of occupational diseases, (2) highlight preferential referral zones and surrounding zones, (3) compare the capture rate from several data sources. For this purpose, the denominator information needed to be refined. The units previously used were most often administrative divisions, but these were not the most accurate ones for this new purpose. In France, data regarding economic activities are given using a specific entity known as the employment area (EA). This entity was created in 1983 by the French National Institute of Statistics and Economic Studies (INSEE), and last updated in 2010 [7]. This subdivision had to be considered when comparing data regarding workers (“denominator”) and their health (“numerator” of occupational diseases).

**Fig. 1:**
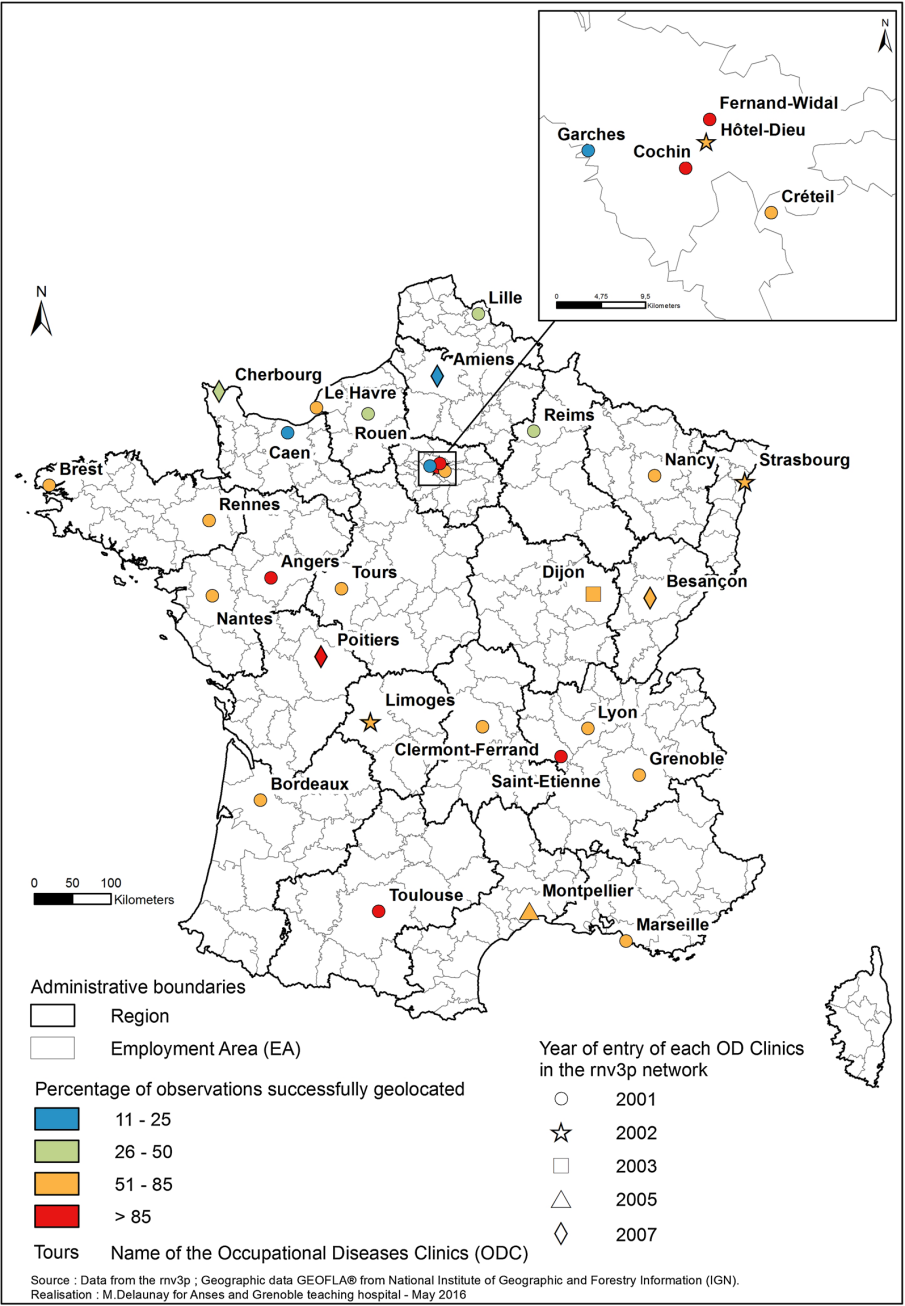
Location of the Occupational Disease Clinics (French National Occupational Diseases Surveillance and Prevention Network, rnv3p)

In France, all 31 Occupational Disease Clinics (OD Clinics) are located in university hospitals and are part of the French National Occupational Diseases Surveillance and Prevention Network (rnv3p) [8] whose main objectives are to describe work situations at risk for specific occupational diseases (OD) and work-related diseases (WRD) (which are not embedded in a list entitling rights for compensation), and to seek new and emerging risks [9]. For more simplicity, WRD and OD are summarised in the “OD” concept in this paper. Analyses of rnv3p network data have been mostly done either on cumulated studies (looking for new rare diseases) [10], or taking the time dimension into account [11]. Apart from the map of the OD Clinics (Fig. 1), and the recent previously cited article [6], no analysis has been done to represent and understand the spatial aspect of rnv3p observations and of the related variables (patients addresses, workplace addresses, and the referring physician’s address).

The aim of the present study was to consider the French National Occupational Diseases Surveillance and Prevention Network as a spatial object and to describe its catchment (preferential catchment areas, surrounding zones). To achieve this, we defined three stages. The first was to describe the rnv3p network and the data it collected. The second was to analyse the rnv3p data with reference to the underlying workforce (denominator) in the employment areas. The third stage was to analyse rnv3p catchment of a well-known occupational disease (mesothelioma), in reference a data source thought to be exhaustive.

## Methods

**Fig. 2:**
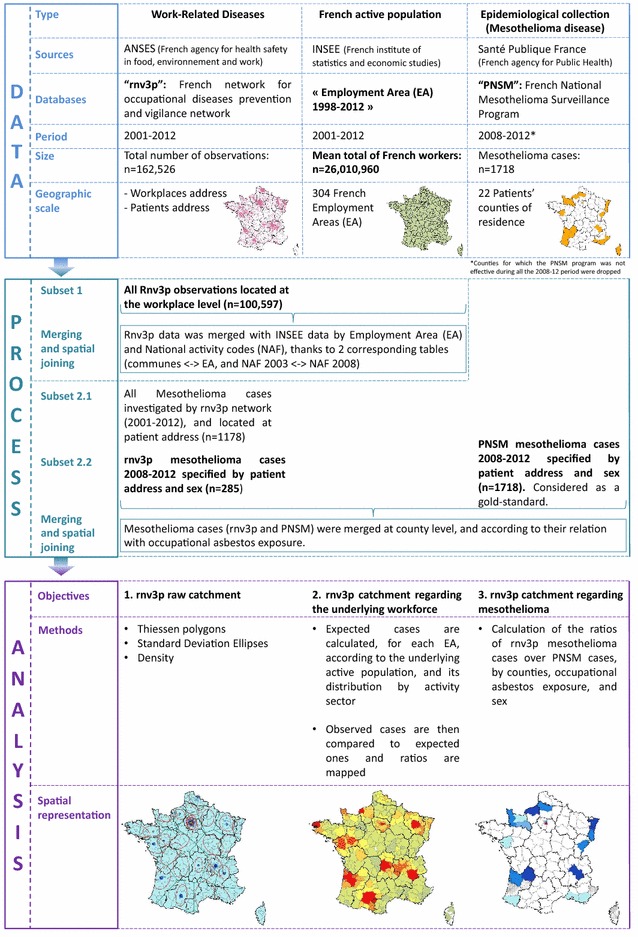
Summarized methodology for the highlightment of French OD clinics preferential recruitment zones. *rnv3p* French National Occupational Diseases Surveillance and Prevention Network, *INSEE* French National Institute of Statistics and Economic Studies

The whole study process is described in Fig. 2, detailing the three data sources, the periods, geographic scales and subsets considered, and the methods used to answer the 3 objectives.

In order to meet our first objective (an approximate description of rnv3p catchment), we studied the rnv3p observations for the 2001–2012 period at the level of each workplace considered as “responsible” for at least one case of an occupational disease, and then localized it according to municipality. OD Clinics collect four main categories of data, presented in four different tables: “Patient” (anonymized identification codes, age, sex, and address), “Workplace” (anonymized identification code, activity sector, and address), “Consultation” (date of consultation, referring physician and their address, etc.) and “Problem” (disease, exposures and their degree of imputability with respect to the disease, occupation, work station, etc.). For information, the workplace is rated as “responsible” of the disease, if the level of attributability assessed by the OD specialist physician is high, taking into account the type of disease, partient’s past medical history, patient’s history of occupational exposures (nature, duration), estimated levels of exposures, the chronology of the appearance of the disease, and previous medical knowledge. These tables are summarized in a single table of “Observations”, available nationally, without any possibility of direct identification of the patient or their workplace. Nevertheless, this table, which is usually used for data analysis, includes patient and workplace codes. We choose to localize the observations at the address of the workplace rather than to the patient’s address, as this was of greater interest in terms of surveillance and subsequent prevention. To match the address of the workplace to the observation, we had to consecutively link the different tables. Firstly, “Observations” and “Patients” were merged in order to extract the workplace identity codes. Secondly, “Observations” and “Workplace” were merged to find the addresses of the workplaces (postal code and city). When the observation was enriched with location information, we used a corresponding table [12] that matched the postal code and city with the name of the municipality.

Three main tools were used to describe the rnv3p data: density, standard deviation ellipse and Thiessen polygons [13]. Rather than simply mapping cases with points, the density is a raster which depicts the concentration of cases, and allows a better visualization of their distribution. Our chosen representation corresponds to a raster with 500 × 500 grid size which ignores administrative boundaries. Since data were located by municipality, a core density tool was applied, the weighting being the number of superposed observations in the same location. From each observation point, the density tool applied a concentric search for other observations within a 10 km radius. Standard deviation ellipses (SDE) were used to summarize the central tendency, dispersion and directional trends of observations made by each OD Clinic. They identify an area comprising 68 % of their information. For this reason, ellipses only give relative information, without any information on the actual number of total observations seen by each clinic. Ellipses might have a larger radius for OD Clinics that have a low number of observations if some of these are located far away from the clinic. In order to include quantitative information useful for interpretation on some of the maps we have added circles proportional to the number of observations recorded by each OD Clinic. Thiessen polygons are a way of representing a purely theoretical preferential recruitment area around clinics. They are totally independent of the real spatial distribution of the observations. They are generated from a set of points (here all OD Clinics) so that any location inside the polygon is closer to that point than any of the other sample points [13].

To achieve our second objective (description of rnv3p data in terms of the underlying workforce), we used data from INSEE on the distribution of five main activity sectors (agriculture, construction, industry, commerce and non-commercial services) at the employment area level (Metropolitan France being partitioned in 304 “employment areas”). The first step was to update the national activity codes in the rnv3p observation tables in order to be comparable to the denominator of the data source. As the INSEE data used the 2008 national activity codes (NAF code) whereas the rnv3p data used the 2003 NAF codes for the period (2001–2012), a merged table [14] between NAF 2003 and 2008 codes was used. The distribution of communes within the 304 employment areas was then used [7]. The second step was to combine data belonging to the same employment areas, in order first to represent the rate of OD per 10,000 workers. Finally, a rate based on the ratio between numbers of rnv3p observed OD and expected OD was calculated for each employment area. The expected number of observations is an estimate taking into account the number of employees by employment area *for each activity sector* and the average rate of diseases attributed to this sector by the rnv3p nationally. Assuming a homogeneous distribution of disease within the same activity sector, for each of the five sectors considered this indicator gave a rate of OD by employment area. To calculate a national OD rate for each sector, we took into account: the total number of OD per sector, the average number of employees in this sector for the period concerned (12 years, from 2001 to 2012). This national OD rate was then applied to each geographic unit. Depending on the number of employees, the rate calculated the number of expected OD, which enabled us to identify areas with “over” and “under” detection of OD in the rnv3p network. To express this rate, a classification according to standard deviation was chosen. Indeed, this allows one to show the difference between the rate found for each employment area and the average of the rate, by expressing this difference in terms of fractions of the standard deviation of the mean value. Class interruptions were taken with equal ranges and proportional to standard deviation.

Our third objective was to compare the rnv3p data with that from another data source for a given occupational disease. As an example, we chose mesothelioma for the following reasons: the French National Mesothelioma Surveillance Program (PNSM) is a large scale epidemiological surveillance system based on some French counties (“départements”) [15, 16] and this disease is mostly due to occupational exposure to asbestos [17]. PNSM data were only available for the patients’ location and were merged by county. We consider the counties for which the PNSM was still active in 2012 (n = 22), and made the comparison with rnv3p for a period of 5 consecutive years (2008–2012). Under the hypothesis that the PNSM identification of mesothelioma cases is exhaustive on these departments, we estimated the rnv3p catchment rate of this disease by counties. Cases related to either para-occupational exposure or environmental exposures were excluded. While PNSM only identifies pleural mesothelioma, it was not possible to automatically differentiate pleural from peritoneal mesothelioma in the rnv3p data as they are indexed using the same ICD10 code. For this reason, all rnv3p mesothelioma cases were considered. Nevertheless, as the proportion of peritoneal cases is low (8 %, [18]), it was assumed this would not significantly alter the comparison of capture rates between the different OD Clinics.

Two analyses were conducted successively. The first one compared the catchment of all mesothelioma (at patients’ addresses) by the two sources rnv3p and PNSM (subset 2.2 mentioned in Fig. 2). Secondly, only cases for which occupational asbestos exposure was considered as the probable cause of the disease were analysed for both rnv3p and PNSM. Regarding PNSM, only 75 % of the men and 76 % of the women could undergo an enquiry to determine asbestos exposure (with differences across départements). It was assumed that, at the department level, the proportion of occupationally related mesothelioma was equivalent in the patients that could be interviewed, and in the remaining ones. This was modelled as follows. For instance, for the Manche Département, 48 mesothelioma cases were identified by PNSM among males, 21 of them (44 %) undergo an enquiry regarding asbestos exposure, of which 18 (86 %) were considered as related to occupational asbestos exposure; 27 could not be interviewed. The total expected number of mesothelioma cases related to occupational asbestos exposure according to PNSM in the Manche département was calculated as the sum of the ones with a positive result of the enquiry (n = 18), and of the number of mesothelioma expected to be related to occupation in the 27 remaining subjects (n = 27 × 0.86 = 23), which means a total of n = 41.

## Results

Regarding the geocoding process for the Observation table, information has been lost at two successive levels. Firstly when merging the “Observation” and “Patient” tables: only 110,578 of the 162,526 observations could be linked to a workplace. This is partly due to the fact that for retired people who came for the investigation of chronic diseases (such as cancers), it was not possible to record previous company where they were exposed. The second one was in matching the municipality (commune, the smallest French administrative division) address: only 100,597 out of 110,578 observations could be matched. Table 1 summarizes the information lost in the three tables of interest (Patient, Workplace and Observations) and indicates the rate of concordance for each of them.

**Table 1:** Number of geolocated addresses for rnv3p “Observations”, “Patient” and “Entreprises” tables (rnv3p 2001–2012)

rnv3p main tables (number of observations)	Observations n = 162,526	Patients n = 192,281	Workplaces n = 70,916
No link with the workplace (n=)	51,948		
Link with workplace (n=)	110,578		
Address not recorded (n=)	8667	24,002	4980
Address not found during the geolocalisation process (n=)	1314	16,032	2262
Match rate^a^ (%)	98.7	90.5	96.6
Total number of rnv3p observations located with reference to the primary rnv3p tables (n=)	100,597	152,247	63,674
Percentage of rnv3p observations successfully geolocated	61.9	79.1	89.8

^a^Match rate refers to the percentage of addresses successfully geolocated with reference to the only entries that have one address informed

Since the geocoding process was based on matching tables in which geographic information was already listed, 98.7 % of our data (n = 100,597) linked to a workplace and having an address were successfully geolocated. This means they had a score of 100 indicating a perfect match, an A match (automatically matched) and an M status (the address is matched). Nevertheless, there are important differences in the percentage of geolocated observations between the different OD clinics (from 11 to 93 %) (Table 2). This information should be borne in mind when interpreting the maps.

**Table 2:** Geolocated observations for each French OD clinic (rnv3p 2001–2012)

OD Clinics	Total of observations	Observations linked to the workplace	Observations geolocated
Amiens	564	213	122 (22 %)
Angers	2652	2456	2386 (90 %)
Besançon	487	342	277 (57 %)
Bordeaux	14,269	10,639	9626 (67 %)
Brest	5514	3239	3118 (57 %)
Caen	6322	956	723 (11 %)
Cherbourg	2121	754	551 (26 %)
Clermont	3425	2997	2942 (85 %)
Cochin	13,790	12,548	12,365 (90 %)
Créteil	12,194	7759	7519 (62 %)
Dijon	564	485	459 (81 %)
Fernand	5921	5582	5471 (92 %)
Garches	10,082	2190	1816 (18 %)
Grenoble	7668	4928	4854 (63 %)
Hôtel-Dieu	752	661	544 (72 %)
Le Havre	4557	2576	2442 (54 %)
Lille	10,212	5306	3844 (38 %)
Limoges	291	194	179 (62 %)
Lyon	12,751	7848	7464 (58 %)
Marseille	2121	1765	1573 (74 %)
Montpellier	861	603	560 (65 %)
Nancy	6129	5599	4725 (77 %)
Nantes	9902	8876	5666 (57 %)
Poitiers	894	834	814 (91 %)
Reims	3227	1268	1151 (36 %)
Rennes	1561	1194	1182 (76 %)
Rouen	4573	2245	2121 (46 %)
Saint-Etienne	2369	2219	2127 (90 %)
Strasbourg	3641	2536	2431 (67 %)
Toulouse	11,176	10,487	10,392 (93 %)
Tours	1936	1279	1153 (60 %)
Total	162,526	110,578	100,597 (62 %)

**Fig. 3:**
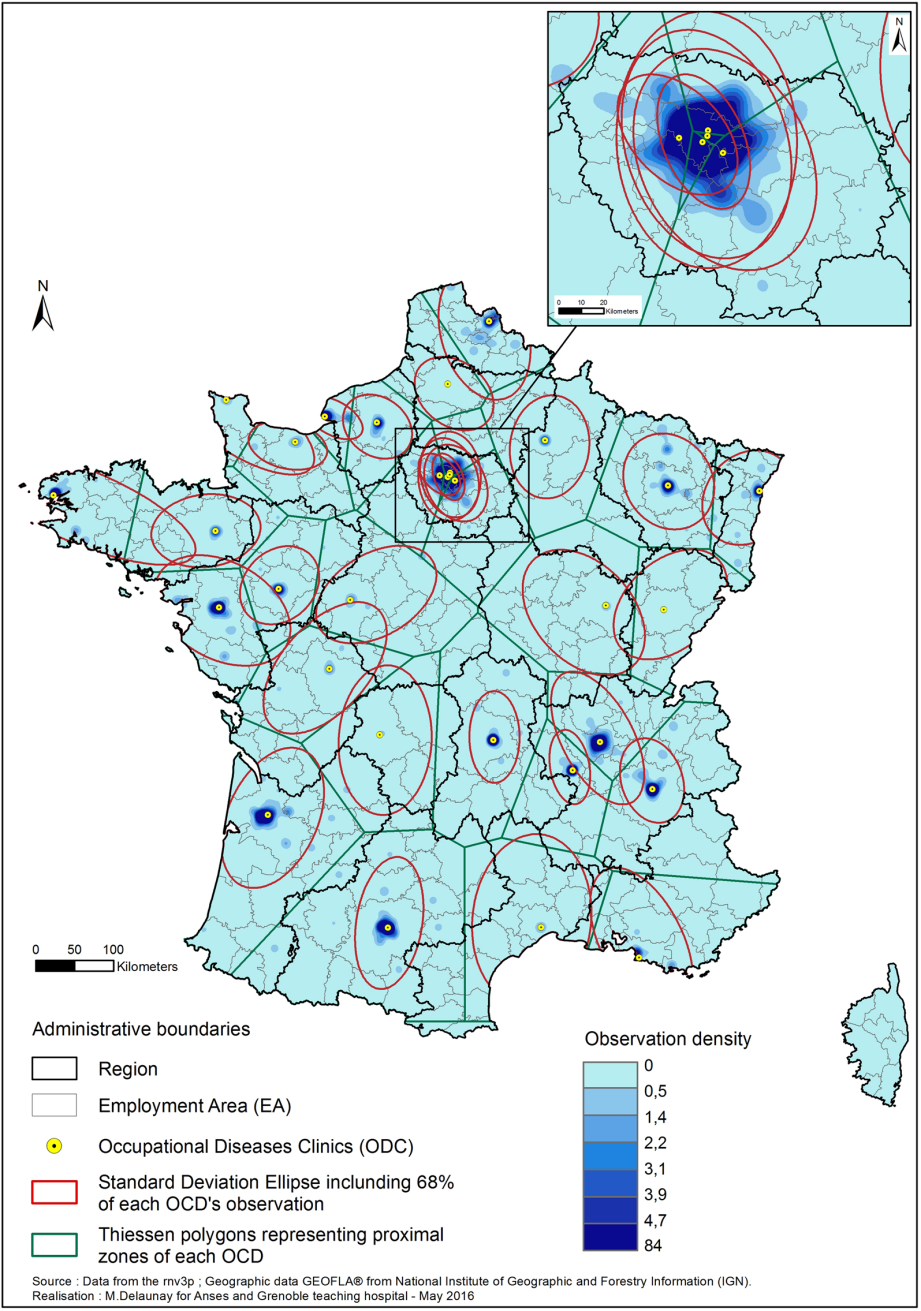
Spatial distribution of rnv3p OD Clinics observations. *OD clinics* Occupational Diseases Clinics. The density is represented by a *blue scale*, directional distribution are appreciated by standard deviation ellipses (*red*), and theoretical recruitment area based on geometric calculation through Thiessen polygons (*green*)

The 100,597 observations successfully geocoded to the workplace address, mapped by the three following methods (density method, standard deviations ellipses, and Thiessen Polygons) are presented together on Fig. 3. At a first glance, this figure shows an almost complete coverage of the country. Ellipses also show a satisfying distribution of the catchment areas throughout the country. These ellipses are quite similar to the theoretically expected catchment areas shown by the Thiessen polygons (“ideal” geometric catchment). When two OD Clinics are close (e.g. Dijon and Besançon in eastern France), ellipses are not centered on the clinic, but shifted away from the nearby OD clinic, which also corresponds to the region it belongs to. Regarding Paris region, which has five OD Clinics (Paris Cochin, Hôtel-Dieu, Fernand-Widal, Garches and Créteil), there is superposition of the catchment areas. Secondly, the map clearly highlights that observations are mainly located in cities with OD Clinics and their surrounding areas: ellipses are centred on the OD Clinics, with the highest density of observations at their centre. The zones of few observations are those in between ellipses, which mostly correspond to the peripheral zones of the Thiessen polygons.

**Fig. 4:**
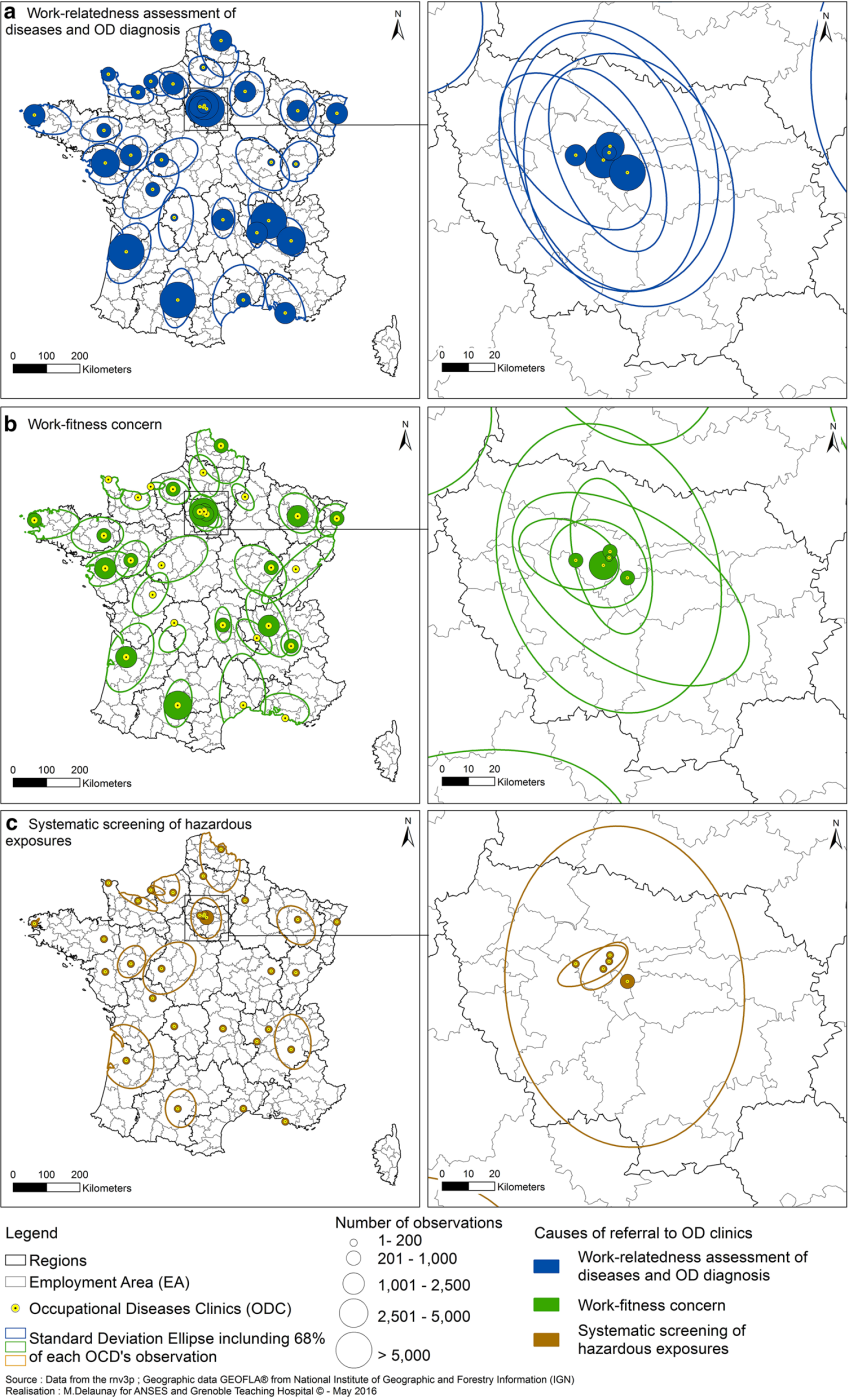
Catchment areas of OD Clinics according to the main cause of patient referral: work-relatedness assessement of diseases and diagnosis of OD (**a**), work-fitness concern (**b**), systematic screening of hazardous exposures (**c**). *Circles* are proportional to the number of observations, and standard deviation ellipses show the main geographical catchment area for each OD clinic. *OD clinics* occupational diseases clinics

The SDE representation was then used to distinguish spatial observation areas according to the three main causes of referral to OD Clinics (Fig. 4): work-related assessment of disease and OD diagnosis, work-fitness concerns, systematic screening of exposure to hazardous substances for a limited number of diseases in hospitalized patients (usually lung cancers, but the type of disease screened might vary according to OD Clinic and time period). The assessment of a disease as being work-related or a clear OD diagnosis were the main reasons for referring patients to OD Clinics. At the single OD clinic level, we can see some highly specific situations, such as that of Limoges (west centre France), based on a relatively small number of geolocated observations (n = 179/total = 291). The ellipse for work-fitness problems is shifted to the south of the city as the problems were mostly referred to the OD Clinics by occupational physicians employed by companies located south of Limoges (Fig. 4b). A completely different example is that of referral for work-related disease in the Rhône-Alpes region. Until 2012 patients seen in Grenoble OD Clinic for systematic hazardous substances assessment came from workplaces in a more extended area than for the other reasons of referral. As Table 3 shows, for 16 OD Clinics, the ellipse size is relatively large for the assessment of the work-relatedness of diseases and OD diagnosis. For 13 OD Clinics, the ellipse areas for work-fitness concerns are relatively large. For 2 OD Clinics (Creteil and Grenoble), ellipse size is greatest for systematic screening of hazardous exposures. Regarding referral to OD Clinics in Paris and its surrounding area (called the “Ile de France”), the “systematic screening of exposure to hazardous substances” issue is the only reason for referral that displays a very large differences in capture areas. This is because the Creteil OD Clinic is a specialist centre in the systematic screening of patients presenting with lung cancer (Fig. 4c).

**Table 3:** Ellipse size (in km^2^) accounting for 68 % of observations around each OD Clinic according to the main cause of patient referral

OD Clinics	Observations		Assessment of work-relatedness of diseases and OD diagnosis		Work-fitness concerns		Systematic screening of exposure to hazardous substances	
	Number	Ellipse size (km^2^)	Number	Ellipse size (km^2^)	Number	Ellipse size (km^2^)	Number	Ellipse size (km^2^)
Amiens	122	11,818	107	7262	10	8594	0	–
Angers	2386	7848	1279	7988	314	8503	73	6192
Besançon	277	14,269	200	10,442	50	16,475	0	–
Bordeaux	9626	16,945	7313	17,222	1083	21,267	143	20,457
Brest	3118	15,051	2233	8209	213	9955	5	87
Caen	723	4385	422	4518	122	3871	9	2546
Cherbourg	551	7392	366	4801	13	2674	1	–
Clermont-Ferrand	2942	5987	2318	6590	555	4046	0	–
Cochin	12,365	6083	7754	5216	3470	7267	7	208
Créteil	7519	6205	5797	5934	410	4444	233	8702
Dijon	459	18,421	192	17,964	262	17,531	0	–
Fernand-Widal	5471	7272	4233	6610	492	1359	0	–
Garches	1816	2033	1340	2286	232	769	0	–
Grenoble	4854	6889	3427	7542	811	3656	108	14,015
Hôtel-Dieu	544	1794	349	2159	105	952	6	237
Le Havre	2442	2936	928	3604	58	766	95	891
Lille	3844	11,245	1908	12,023	663	14,232	145	13,823
Limoges	179	19,141	156	15,367	9	4914	1	–
Lyon	7464	13,484	5547	12,641	1521	16,572	8	–
Marseille	1573	11,705	1263	10,293	100	8937	0	–
Montpellier	560	19,233	451	17,823	92	20,667	0	–
Nancy	4725	11,669	2218	10,424	1702	10,956	37	10,104
Nantes	5666	16,799	4001	12,854	1163	9809	0	
Poitiers	814	17,965	640	20,041	90	9418	2	
Reims	1151	10,956	1037	11,042	70	3430	3	
Rennes	1182	10,260	307	7122	530	13,191	1	
Rouen	2121	5606	1140	5344	662	5330	11	5073
Saint-Etienne	2127	4074	1698	3649	181	7342	9	
Strasbourg	2431	9225	1196	9685	512	5527	1	
Toulouse	10,392	11,621	5327	12,517	3776	9094	49	8359
Tours	1153	14,552	780	12,609	114	18,492	125	18,356
Total	100,597	311,994	65,927	291,801	19,385	270,067	1072	109,053

Metropolitan France area = 551,500 km^2^

**Fig. 5:**
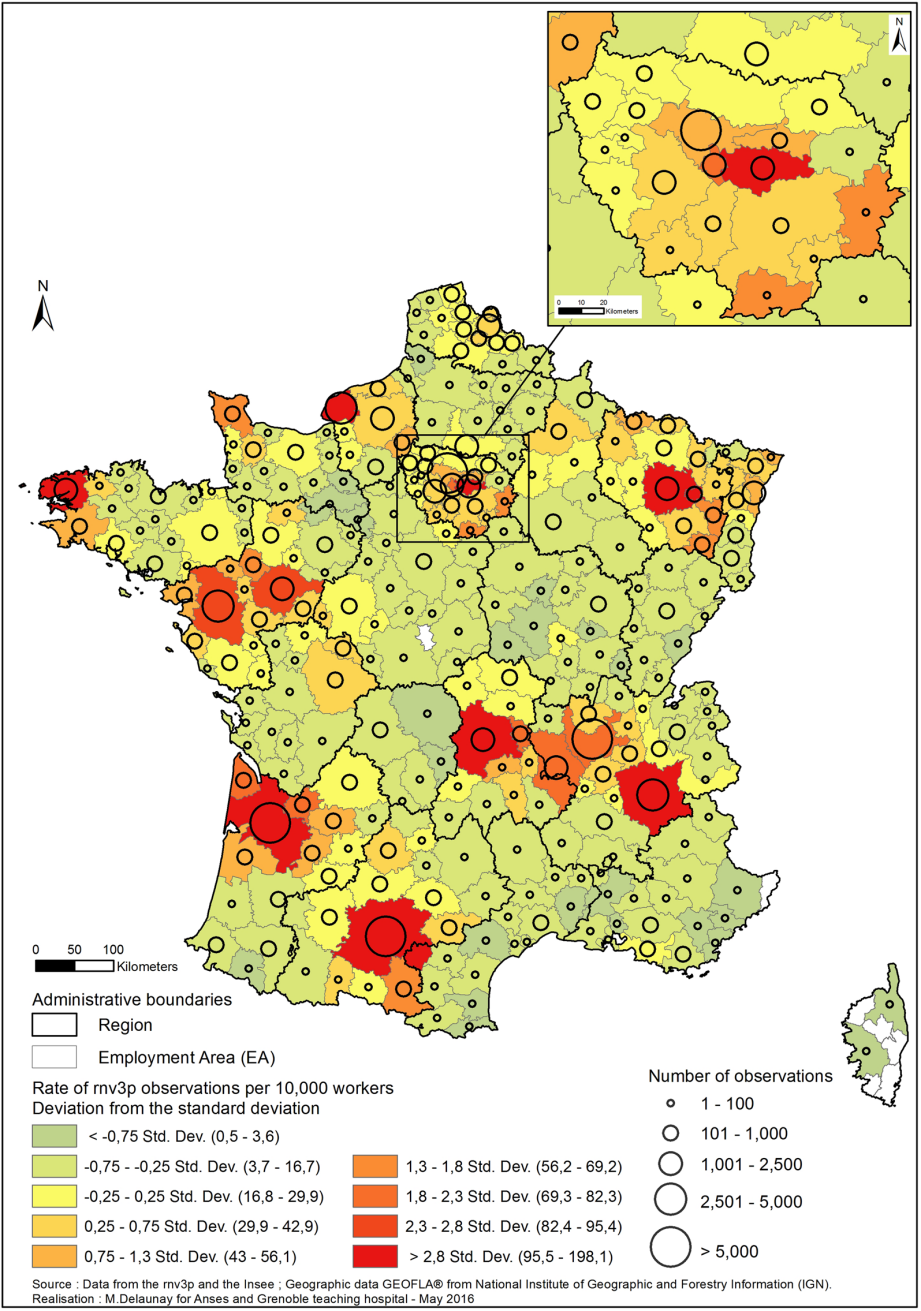
Rate of rnv3p observations per 10,000 workers from all activity sectors (employment areas level). *Sources* rnv3p data 2001–2012; INSEE data for the average number of active workers on the same period (INSEE: French National Institute of Statistics and Economic Studies)

In order to take into account the fact that the main cities and suburbs are associated with the greatest density of active workers, Fig. 5 describe the catchment areas taking into account the number of active workers in each employment area (rate of rnv3p observations with the total number of active workers in the employment area as denominator). 297 of the 304 employment area had cases recorded within rnv3p. There were no rnv3p observations for seven employment areas of which five are located in the island of Corsica that has no OD Clinic. The two other areas for which there were no observation are “Issoudun” (central France) and “Menton—Vallée de la Roya” (south-eastern France, at the Italian border) that had an average number of active workers in the 2001–2012 period of 9319 and 20,221 respectively. Overall, the mean number of OD per 10,000 workers catched by OD clinics for the period considered is 23,4/10,000 workers. Nevertheless, variance is very important (standard deviation = 26, minimum = 0.5, maximum = 198). The nine employment areas corresponding to the highest part of the distribution (>2.8 deviation standard), are in decreasing order: Lunéville near Nancy (198.2 rnv3p observations per 10,000 workers), Le Havre (rate = 156.9), Toulouse (rate = 149.5), Bordeaux (rate = 142.2), Brest (rate = 118.9), Grenoble (rate = 115.3), Nancy (rate = 109.9), Créteil (rate = 102.3) and Clermont-Ferrand (rate = 97.5). Most of these employment areas coalesce with neighbouring employment areas and show a decreasing rate of observations with increasing distance from the OD clinic. However, employment area with the highest catchment rate are sometimes adjacent to some with the lowest catchment rate (such as Clermont-Ferrand employment zone and the nearby zones of Gueret and Ussel which have capture rate of 2.7 and 3.2 per 10,000 workers). As for Bordeaux, the administrative regional boundary (black line) seems to act like a barrier to referral from the adjacent region. Finally, where ellipses overlap (employment areas with two OD Clinics) there does not appear to be better capture of observations. In fact, these employment areas often show an observation rate which is intermediate between those of the nearby cities in which OD Clinics are located (e.g. Angers-Nantes; Lyon-Grenoble).

**Fig. 6:**
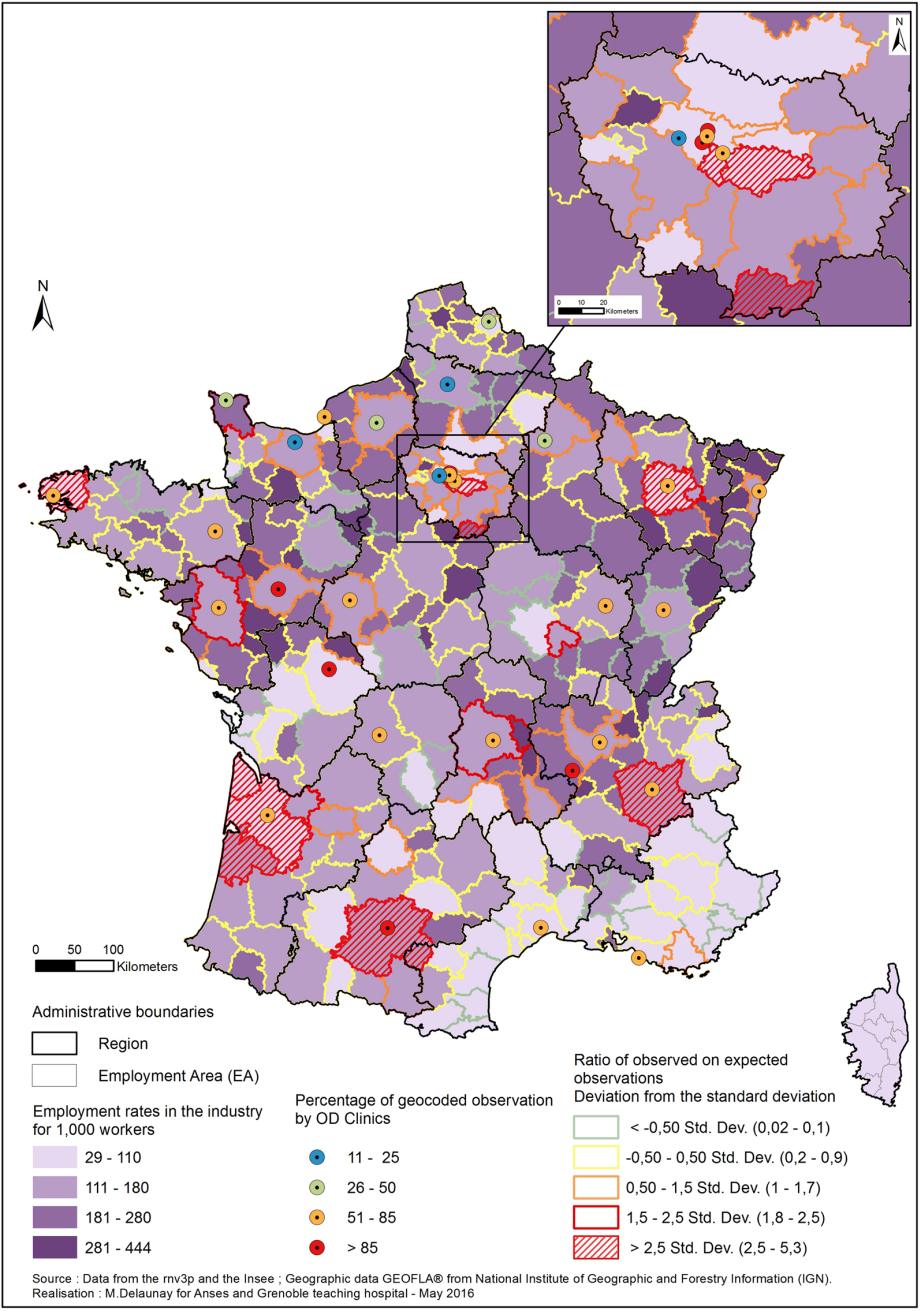
Distribution of Industrial workers in France, and employment area with higher catchment of OD by OD clinics network. *OD* occupational diseases. Employment rates in the industrial sectors are shown by depth of *purple* (at the employment areas level), and occupational diseases (OD) reported to the rnv3p by OD Clinics (number of observations) are reported to the expected number (*coloured boundaries*; highest recruitment zones: *red*-*hatched*). *Sources* rnv3p 2001–2012, and French National Institute of Statistics and Economic Studies (INSEE)

Regarding the industrial sector (manufacturing, mining, chemical industry, metallurgy and metal work etc.), it appears that this activity is more concentrated in the northern half of France, whereas the Mediterranean perimeter has a relatively low percentage of industrial workers (Fig. 6). The majority of the employment areas with the highest concentrations of active industrial workers are small. Here, the number of observed cases over the number of expected ones has been studied to highlight employment areas with more referrals than the average. In general the rnv3p pattern of observation for industrial sectors is in line with what has been previously described, but with some exceptions. The employment area with the highest capture rate of OD among industrial workers are Bordeaux (and some neighbouring zones), Toulouse, Grenoble, Nancy, Brest and three zones in Ile de France region. All these employment area have an OD clinic. We notice that, except for Toulouse, none of these OD clinics belong to the highest class regarding the percentage of geocoded information (the information mapped doesn’t only translate the amount of available information). None of these employment areas belong to those having the highest proportion of industrial workers; six of them are even in the lowest quintile.

Finally, in order to take into account the five broad categories of occupational activities (agriculture, construction, industry, commerce and non-commercial services), the ratios of observed versus expected OD for each category of activity was calculated and mapped for each employment area. This map (Additional File 1: Fig. S1) was exactly similar to the one of the rate of OD per employment areas (Fig. 5), which means the pattern of recruitment was not substantially modified by the distribution of activities across employment areas.

Another issue is the type of diseases in a given activity sector that are investigated by the OD Clinics and which could lead to qualitative differences in observations. Table 4 shows the typology of OD attributed to work (according to the first ICD-10 digit) in *industrial sector* for the 11 employment areas with the highest catchment. Overall, there is a predominance of lung diseases (31 %) and psychiatric disorders (19 %), followed by eyes and ears diseases (12 %), dermatological diseases (10 %) and musculoskeletal disorders (10 %), but there are stark differences between OD Clinics, according to the exact nature of the medical supply and expertise they were able to develop.

**Table 4:** Typology of occupational diseases (according to first ICD-10 digit) investigated by the French OD Clinics regarding the industry sector, for the eleven employment areas with the highest catchment

Employment zone	All observations (industry sector)	Observations related to work-relatedness assessment	Subset of observations for which disease is associated to occupational exposure with strong imputability	First digit of the ICD-10 code (main categories)								
				B	C	D	E	F	G	H	I	J
Bordeaux	1627	1173	470		5 %			37 %	4 %	5 %	1 %	20 %
Brest	537	317	191		3 %			14 %	3 %	5 %		53 %
Créteil	288	214	143		2 %			13 %	3 %	8 %	1 %	40 %
Grenoble	1250	937	572		5 %	1 %		5 %	1 %	13 %	1 %	45 %
La Teste-de-Buch	84	50	20					20 %		5 %		55 %
Le Havre	1225	499	165		5 %	1 %		2 %		5 %	1 %	75 %
Nancy	659	303	173		6 %	6 %		14 %	2 %	4 %		29 %
Nemours	54	36	17		6 %	6 %	12 %	6 %		6 %		47 %
Orly	328	243	130	1 %	6 %	1 %		22 %	4 %	10 %		29 %
Pauillac	17	15	6					33 %				17 %
Toulouse	1610	1021	774					25 %	5 %	21 %		10 %
Total	7679	4808	2661	2	89	24	3	500	86	310	13	821

Employment zone	All observations (industry sector)	Observations related to work-relatedness assessment	Subset of observations for which disease is associated to occupational exposure with strong imputability	First digit of the ICD-10 code (main categories)								
				K	L	M	N	R	S	T	U	Z
Bordeaux	1627	1173	470		8 %	14 %		2 %		2 %	1 %	
Brest	537	317	191		10 %	2 %		2 %		1 %	1 %	8 %
Créteil	288	214	143	1 %	12 %	13 %		4 %		1 %	1 %	
Grenoble	1250	937	572	1 %	10 %	5 %		6 %		1 %	3 %	2 %
La Teste-de-Buch	84	50	20		5 %	15 %						
Le Havre	1225	499	165		5 %	1 %	1 %	1 %		1 %	1 %	2 %
Nancy	659	303	173		17 %	11 %		1 %		2 %	3 %	4 %
Nemours	54	36	17		6 %	6 %						6 %
Orly	328	243	130		12 %	6 %	1 %	2 %		3 %	3 %	
Pauillac	17	15	6		33 %					17 %		
Toulouse	1610	1021	774		11 %	16 %		3 %		3 %		6 %
Total	7679	4808	2661	8	274	272	4	86	2	46	39	82

Percentages are presented by lines. A and B: certain infectious and parasitic diseases; C: neoplasms; D: diseases of the blood and blood-forming organs and certain disorders involving the immune system; E: endocrine, nutritional and metabolic diseases; F: mental and behavioural disorders; G: diseases of the nervous system; H: diseases of the eye, adnexa, ear and mastoid process; I: diseases of the circulatory system; J: diseases of the respiratory system; K: diseases of the digestive system; L: diseases of the skin and subcutaneous tissue; M: diseases of the musculoskeletal system and connective tissue; N: diseases of the genitourinary system; R: symptoms, signs and abnormal clinical and laboratory findings, not elsewhere classified; S and T: injury, poisoning and certain other consequences of external causes; U: codes for special purposes; Z: factors influencing health status and contact with health services

**Fig. 7:**
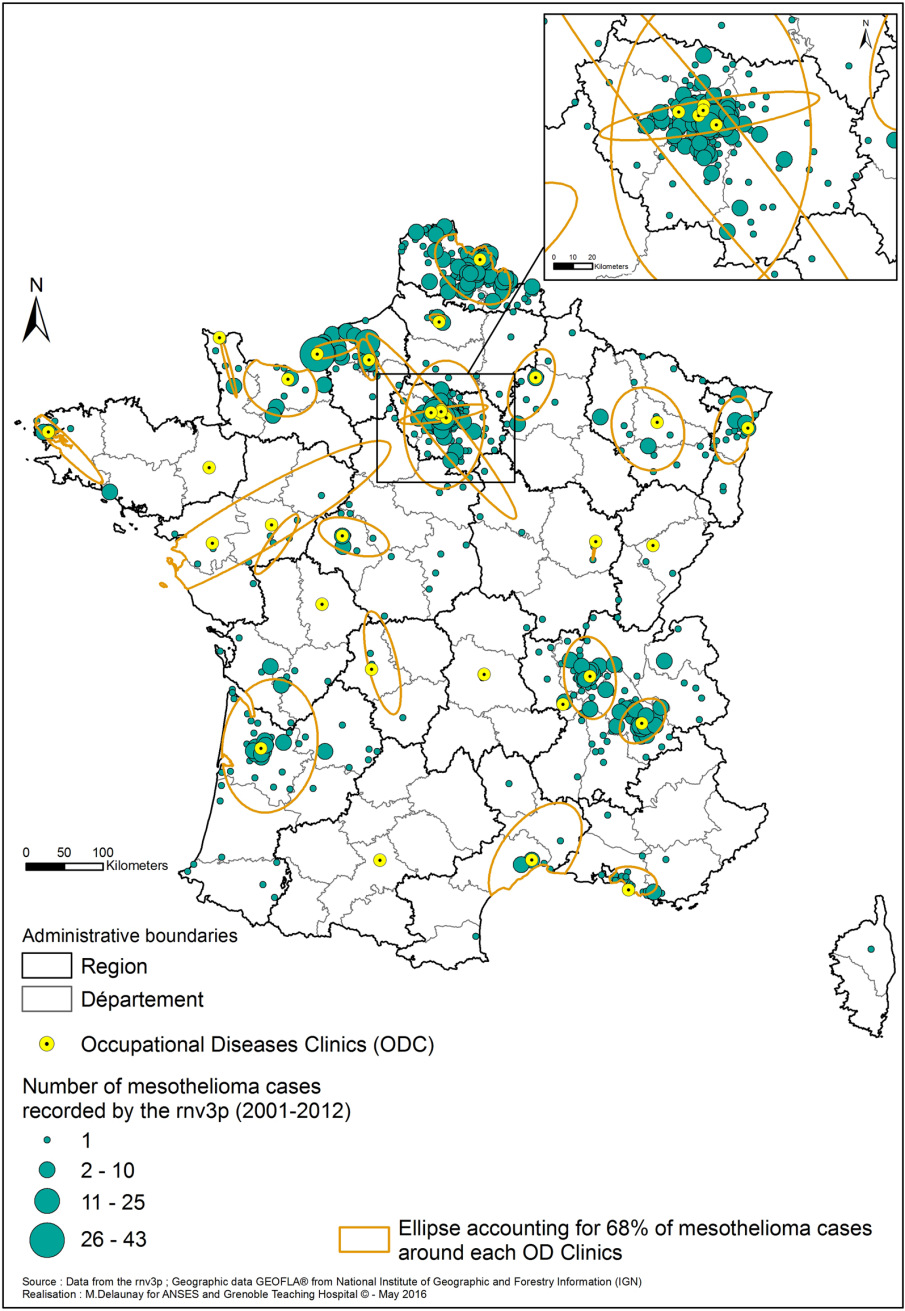
Mesothelioma cases recorded by the rnv3p at the “département” level for the period 2001–2012

Regarding mesothelioma, we first mapped the 1178 cases recorded by the rnv3p for the 2001–2012 period at the county (département) level (Fig. 7). Most of the cases investigated in OD clinics came from 11 of the 96 “départements” of mainland France. Most of them are located in the Northern part of France (Seine Maritime where Rouen OD clinic is located, Nord and Pas de Calais mostly captured by the Lille OD clinic, and cases from the Ile de France region captured by OD clinics in the Paris area). Three “départements” with a relatively high number of mesothelioma cases recorded by the rnv3p are located in the southern half of France (Gironde by the Bordeaux OD clinic, and Isère and Rhône in the Rhône-Alpes region, by the Grenoble and Lyon OD clinics).

We then compared, for the same 2008–2012 period, the number of all mesothelioma cases recorded by the epidemiological source considered as the gold standard (PNSM, total = 1718) and by the rnv3p (total = 285 cases), by “département” and sex (Table 5). Both sources had a sex-ratio of 0.3 (number of females’ cases over number of males’ cases). In 5 départements out of 22, no males’ cases have been reported in rnv3p, whereas the inverse situation is encountered in 13 départements. The global rnv3p catchment rate for this disease (PNSM taking as a reference) is 17 % for males and 15 % for females, with strong variations between counties showing a strong OD clinic effect (from 0 to 44 and 59 % respectively for females’ and males’ cases, with the highest value associated to Val de Marne). For each clinic, there is usually a wide discrepancy between the capture rates of males’ cases and females’ cases. Usually the capture rate of female’s cases for all mesothelioma, is lower than the one for males’ cases, except for 3 départements: Gironde (34 % for women vs 19 % for men), Seine-Saint-Denis (21 vs 11 %), and Calvados (24 and 3 % respectively, but based on a lower number of cases). Then, the rnv3p mesothelioma capture rate was assessed considering only the cases for which an occupational asbestos exposure was certified and considered as the probable cause of the disease (Table 6 for males and Table 7 for females). Regarding males’ cases, the catchment rate did not change globally (191/1107 = 17 %), and the variations in each OD clinics were very limited. Nevertheless, considering women’s cases, the catchment rate raised from 15 to 47 % when considering the subset of cases for which the disease was attributed to occupational asbestos exposure (24 cases for rnv3p, and 51 for PNSM). In Calvados 5 cases have been captured by rnv3p over 7 cases for PNSM. Moreover, in Isère and Val-de-Marne, more cases have been related to an occupational exposure to asbestos within rnv3p than within the PNSM (n = 6 vs 4, and 4 vs 3 respectively). We got the confirmation by checking the medical reports that these female cases were occupationally related, and not para-occupational. Again, these analyses show a strong OD clinic effect.

**Table 5:** All mesothelioma cases identified by PNSM and rnv3p for the 2008–2012 period, by “département” and sex

Counties identification (number and name of French «départements»)	All Mésothélioma (2008–2012)										
	PNSM				rnv3p				rnv3p catchment rate (%)		
	M	F	Total	Sex-ratio	M	F	Total	Sex-ratio	M	F	Total
06—Alpes Maritimes	60	32	92	0,5	0	0	0	–	0	0	0
13—Bouches-du-Rhône	182	40	222	0,2	2	0	2	0	1	0	1
14—Calvados	37	25	62	0,7	1	6	7	6	3	24	11
24—Dordogne	19	14	33	0,7	6	0	6	0	32	0	18
25—Doubs	14	3	17	0,2	2	0	2	0	14	0	12
2A—Haute-Corse	5	1	6	0,2	0	0	0	–	0	0	0
2B—Corse du Sud	8	2	10	0,3	0	0	0	–	0	0	0
33—Gironde	103	32	135	0,3	20	11	31	0,6	19	34	23
38—Isère	95	28	123	0,3	34	9	43	0,3	36	32	35
40—Landes	23	9	32	0,4	0	0	0	–	0	0	0
44—Loire-Atlantique	133	24	157	0,2	1	0	1	0	1	0	1
47—Lot-et-Garonne	12	3	15	0,3	0	0	0	–	0	0	0
50—Manche	48	13	61	0,3	5	0	5	0	10	0	8
61—Orne	26	7	33	0,3	2	0	2	0	8	0	6
64—Pyrénées-Atlantiques	34	11	45	0,3	1	1	2	1	3	9	4
67—Bas-Rhin	43	11	54	0,3	18	2	20	0,1	42	18	37
68—Haut-Rhin	23	5	28	0,2	3	0	3	0,0	13	0	11
76—Seine-Maritime	161	57	218	0,4	74	12	86	0,2	46	21	39
80—Somme	23	9	32	0,4	5	1	6	0,2	22	11	19
83—Var	126	26	152	0,2	1	0	1	0,0	1	0	1
93—Seine-Saint-Denis	64	24	88	0,4	7	5	12	0,7	11	21	14
94—Val-de-Marne	71	32	103	0,5	42	14	56	0,3	59	44	54
Total	1310	408	1718	0,3	224	61	285	0,3	17	15	17

*PNSM* French National Mesothelioma Surveillance Program («Programme National de Surveillance du Mesotheliome»), *Rnv3p* French National Occupational Diseases Surveillance and Prevention Network («Réseau National de Vigilance et de Prévention des Pathologies Professionnelles»), *M* males, *F* Females, *sex*-*ratio* F/M

**Table 6:** Males’ cases of Mesothelioma attributed to occupational asbestos exposure, for PNSM and rnv3p, by département and sex (2008–2012)

Counties identification (number and name of French «départements»)	rnv3p	PNSM						rnv3p catchment rate of males’ mesothelioma cases attributed to occupational asbestos exposure C = WR_rnv3p_/WR_PNSM_ × 100
	Work-related males cases among rnv3p (WR_rnv3p_)	Total of males cases (T_PNSM_)	Total of cases investigated (T_PNSM-I_)	Work-related cases among T_PNSM-I_ (WR_PNSM-I_) and related proportion (p)	Total of Cases NOT investigated (T_PNSM-NI_)	Expected work-related cases among those not investigated WR_PNSM-NI_ = T_PNSM-NI_ × p	Estimated number of work-related cases WR_PNSM_ = WR_PNSM-I_ + WR_PNSM-NI_	
06—Alpes Maritimes	0	60	45	38 (0.84)	15	13	51	0
13—Bouches-du-Rhône	2	182	140	131 (0.94)	42	39	170	1
14—Calvados	1	37	30	25 (0.83)	7	6	31	3
24—Dordogne	4	19	14	8 (0.57)	5	3	11	37
25—Doubs	1	14	12	9 (0.75)	2	2	11	10
2A—Haute-Corse	0	5	3	2 (0.67)	2	1	3	0
2B—Corse du Sud	0	8	7	6 (0.86)	1	1	7	0
33—Gironde	19	103	88	68 (0.77)	15	12	80	24
38—Isère	28	95	72	63 (0.88)	23	20	83	34
40—Landes	0	23	22	17 (0.77)	1	1	18	0
44—Loire-Atlantique	1	133	60	52 (0.87)	73	63	115	1
47—Lot-et-Garonne	0	12	10	7 (0.70)	2	1	8	0
50—Manche	5	48	21	18 (0.86)	27	23	41	12
61—Orne	2	26	14	12 (0.86)	12	10	22	9
64—Pyrénées-Atlantiques	1	34	28	22 (0.79)	6	5	27	4
67—Bas-Rhin	15	43	39	35 (0.90)	4	4	39	39
68—Haut-Rhin	2	23	14	12 (0.86)	9	8	20	10
76—Seine-Maritime	63	161	127	112 (0.88)	34	30	142	44
80—Somme	4	23	15	12 (0.80)	8	6	18	22
83—Var	1	126	102	91 (0.89)	24	21	112	1
93—Seine-Saint-Denis	6	64	57	40 (0.70)	7	5	45	13
94—Val-de-Marne	36	71	65	52 (0.80)	6	5	57	63
Total	191	1310	985	832 (0.84)	325	275	1107	17

*PNSM* French National Mesothelioma Surveillance Program («Programme National de Surveillance du Mesotheliome»), *Rnv3p* French National Occupational Diseases Surveillance and Prevention Network («Réseau National de Vigilance et de Prévention des Pathologies Professionnelles»), *M* males, *F* females, *sex*-*ratio* F/M, *NA* not attributed

**Table 7:** Females’ cases of Mesothelioma attributed to occupational asbestos exposure, for PNSM and rnv3p, by département and sex (2008–2012)

Counties identification (number and name of French «départements»)	rnv3p	PNSM						rnv3p catchment rate of females’ mesothelioma cases attributed to occupational asbestos exposure C = WR_rnv3p_/WR_PNSM_ × 100
	Work-related females cases among rnv3p (WR_rnv3p_)	Total of females cases (T_PNSM_)	Total of cases investigated (T_PNSM-I_)	Work-related cases among T_PNSM-I_ (n = WR_PNSM-I_) and related proportion (p)	Total of Cases NOT investigated (T_PNSM-NI_)	Expected work-related cases among those not investigated WR_PNSM-NI_ = T_PNSM-NI_ × p	Estimated number of work-related cases WR_PNSM_ = WR_PNSM-I_ + WR_PNSM-NI_	
06—Alpes Maritimes	0	32	20	0 (0.00)	12	0	0	NA
13—Bouches-du-Rhône	0	40	31	0 (0.00)	9	0	0	NA
14—Calvados	5	25	17	5 (0.29)	8	2	7	68
24—Dordogne	0	14	11	1 (0.09)	3	0	1	NA
25—Doubs	0	3	3	0 (0.00)	0	0	0	NA
2A—Haute-Corse	0	1	1	0 (0.00)	0	0	0	NA
2B—Corse du Sud	0	2	1	0 (0.00)	1	0	0	NA
33—Gironde	2	32	29	3 (0.10)	3	0	3	60
38—Isère	6	28	26	4 (0.15)	2	0	4	139
40—Landes	0	9	5	0 (0.00)	4	0	0	NA
44—Loire-Atlantique	0	24	14	3 (0.21)	10	2	5	0
47—Lot-et-Garonne	0	3	2	0 (0.00)	1	0	0	NA
50—Manche	0	13	6	2 (0.33)	7	2	4	0
61—Orne	0	7	7	1 (0.14)	0	0	1	0
64—Pyrénées-Atlantiques	0	11	8	0 (0.00)	3	0	0	NA
67—Bas-Rhin	1	11	10	1 (0.10)	1	0	1	91
68—Haut-Rhin	0	5	1	0 (0.00)	4	0	0	NA
76—Seine-Maritime	5	57	43	11 (0.26)	14	4	15	34
80—Somme	0	9	6	1 (0.17)	3	1	2	0
83—Var	0	26	17	1 (0.06)	9	1	2	0
93—Seine-Saint-Denis	1	24	23	3 (0.13)	1	0	3	32
94—Val-de-Marne	4	32	31	3 (0.10)	1	0	3	129
Total	24	408	312	39 (0.13)	96	12	51	47

*PNSM* French National Mesothelioma Surveillance Program («Programme National de Surveillance du Mesotheliome»), *Rnv3p* French National Occupational Diseases Surveillance and Prevention Network («Réseau National de Vigilance et de Prévention des Pathologies Professionnelles»), *M* males, *F* females, *sex*-*ratio* F/M, *NA* not attributed

**Fig. 8:**
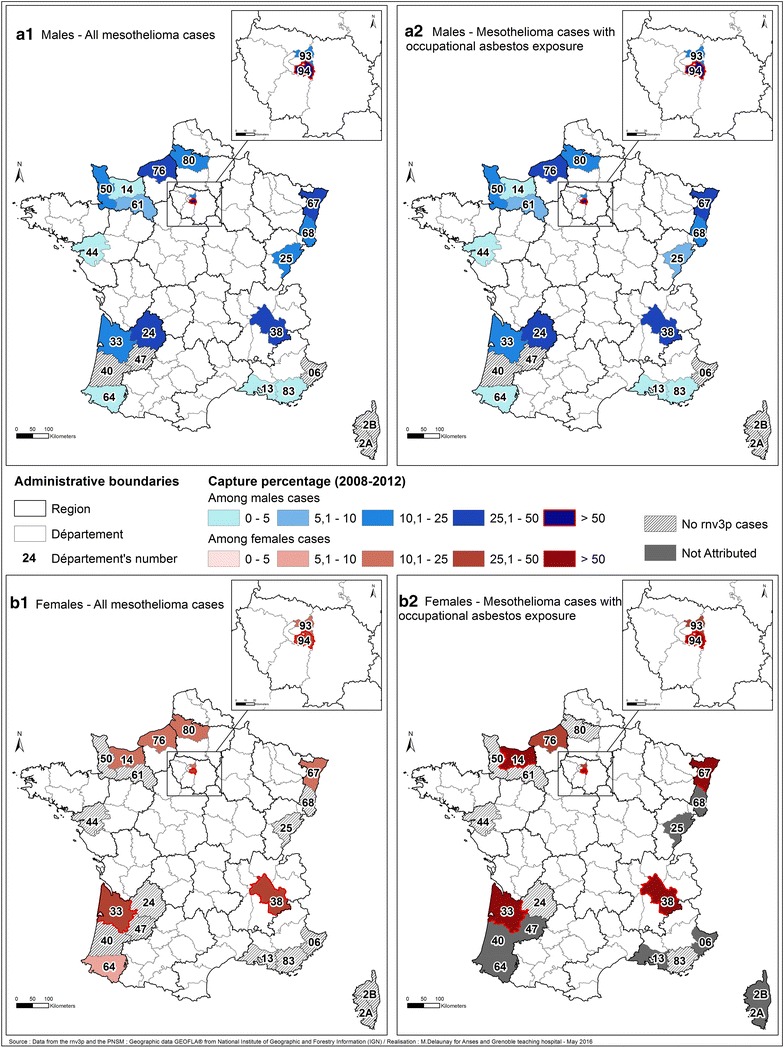
Rnv3p capture rate of mesothelioma cases in comparison with the PNSM, by sex, for all mesothelioma, and for the subset of cases with occupational asbestos exposure (2008–2012). **a1** rnv3p capture rate for all male’s mesothelioma cases. **a2** rnv3p capture rate for male’s mesothelioma cases related to occupational asbestos exposure. **b1** rnv3p capture rate for all female’s mesothelioma cases. **b2** rnv3p capture rate for female’s mesothelioma cases related to occupational asbestos exposure. *Rnv3p* French National Occupational Diseases Surveillance and Prevention Network, *PNSM* French National mesothelioma surveillance program (Programme National de Surveillance du Mésothéliome). The capture rate takes the “NA” value, for départements for which no cases have been identified by PNSM. The comparison is available only for the “départements” covered by the PNSM which are the following: *06*—Alpes Maritimes, *13*—Bouches-du-Rhône, *14*—Calvados, *24*—Dordogne, *25*—Doubs, 2A—Haute-Corse, 2B—Corse du Sud, *33*—Gironde, *38*—Isère, *40*—Landes, *44*—Loire-Atlantique, *47*—Lot-et-Garonne, *50*—Manche, *61*—Orne, *64*—Pyrénées-Atlantiques, *67*—Bas-Rhin, *68*—Haut-Rhin, *76*—Seine-Maritime, *80*—Somme, *83*—Var, *93*—Seine-Saint-Denis, *94*—Val-de-Marne

Figure 8a1, a2, b1, b2 shows the geographical distribution of capture rate of rnv3p compared to PNSM for men and women, for all mesothelioma and the subset of mesothelioma attributed to occupational asbestos exposure. The maps highlight the fact that the capture of mesothelioma cases by rnv3p is very low in the southern counties covered by PNSM, with two notable exceptions, namely Isère where Grenoble OD clinic is located, and Gironde with Bordeaux OD clinic and the nearby Dordogne county. The higher the distance with an OD clinic, the lower is the catchment rate (see for instance Alpes Maritimes at the Italian border) or Corsica. The highest catchments are reached in the Paris area (Val de Marne where the Creteil OD clinic located), Seine Maritime county (where Rouen and Le Havre OD clinics are located), whereas the catchment in the neighboring Normandie counties remains several times lower (Manche where Cherbourg OD clinic is located, Calvados, and Orne). In the Eastern France, the catchement is also 3 times higher in the county where Strasbourg OD clinic is located (Bas Rhin), than in the neigbouring county with no OD clinic. To the contrary some area surrounding OD clinics (such Loire Atlantique with Nantes OD clinic, and Bouches du Rhône with Marseille OD clinic) have a low catchment of mesothelioma cases, as their main activity is dedicated to other medical issues. Figure 8a2, b2 clearly highlights a highest rnv3p capture rate for female’s cases for which occupational asbestos exposure was identified and considered as the probable cause of the disease, in 4 départements.

In conclusion, the catchment by rnv3p of the most emblematic OD through the OD clinics varies across France, according to the proximity of OD clinic, but also to their specialities, and according to sex, and work-relatedness for the subset of females’ cases. Some counties characterised by an important rnv3p recruitment (Nord, Pas de Calais), could not be studied as not covered by the PNSM.

## Discussion

This is the first time that the French OD Clinic network, rnv3p, has been examined from a geographical perspective. The location of OD occurrences (addresses of the workplaces imputed to be sources of disease) has enabled us to describe the catchment zones of OD Clinics, and also of some related surrounding zones. The use of different geographical analysis methods (density, standard deviation ellipses, Thiessen polygons) shows that there is a strong general trend for patients to be referred to OD Clinics in their own employment area or in the neighbouring ones, with a rapid decrease in number of referrals with increasing distance. This attractive force of OD clinics in their neighbouring area might also be related to induced-demand (Roemer’s law [19]), as these zones with teaching hospital correspond to those with the highest provision of health services as shown by the French medical demography atlas [20], except for some of the counties located at the French South border.

We also showed that the “centre effect” can be manifested in different ways, depending on other parameters such as the reason for referral. The catchment was then refined using the size of the underlying active workforce in those “Employment Areas” for which statistical data was available. This revealed some areas associated with a higher capture of cases. We also found a qualitative effect when an OD clinic specialized in a particular type of examination. Taken together, the approach has provided new information to the rnv3p network stakeholders that allows them to better interpret the capture of cases and rnv3p figures. Nevertheless there is still work to do to better describe, within the figures analyzed, what depends from the OD centers’ signatures, from the signature of the underlying economic activity and the related work-related diseases.

The rnv3p data were mapped at the most accurate scale permitted by the availability of the external data sources (“départements” for PNSM, employment areas for INSEE, and municipalities for rnv3p data alone).

The main limitation of our study is the loss of information on observations due to the fact that the “entreprise responsible of the disease” is often a missing data. Indeed, only 59 % of observations were geolocated. There are two main reasons for this. First of all, the “entreprise responsible” could not be recorded when it was not the current one (limitation due to the rnv3p application in its previous version). This affects mainly long latency diseases. Indeed, the percentage of successfully geocoded observations related to a subset of short latency diseases (rhinitis, asthma, contact dermatitis) is 77 %, whereas it is only 24 % for diseases with long latency diseases (cancers, pneumoconioses including asbestosis related pleural plaques). Secondly, the “entreprise address” information was not mandatory, and was filled differently by OD clinics. Even for short latency diseases, there remain differences in the percentages of addresses recorded (Additional File 2: Table S1). From now on, these limitations have been addressed. First of all the new rnv3p information system allows to record previous enterprises, and secondly OD clinics were given the information of the importance to record enterprises addresses, as well as their unique national identifier in order to cross with other databases.

This information had the best quality of geocoding assessed in terms of match rates, match scores, match type and spatial accuracy [21]. This loss of information could induce bias and alter the patterns given by the maps. For this reason, in the legends to the maps we have highlighted the score for geolocation for each OD clinic, so as to alert to caution in interpretation.

Another limitation is that although we highlighted the interaction between the observed catchment and OD Clinic specializations (in terms of emphasis on particular surveillance activities) and the characteristics of the territories in terms of occupational risks; this is still not sufficiently well described and understood at this time. Thus it should not be totally relied on and used to adjust and optimize surveillance missions.

In terms of perspectives, the main challenge is now to design and develop a dynamic cartographic tool, linked to an updated rnv3p database for OD physicians, OH services and stakeholders to enable them to enter and access their data using a systematically mapped and geographic approach. From a technical point of view, the first step will be to put as much emphasis on the coding of geographic information, and identification of the workplace as on the other recorded variables, in order to obtain the most reliable information from the maps. For a better picture of OH, complementary approaches centred on given diseases are needed (e.g. maps of work-related asthma cases and the geographical distribution of their causes). Apart from the specific case of mesothelioma, there are no other surveillance networks which exhaustively cover occupational diseases. Nevertheless, comparisons can be made with some other sources. The most interesting one is the salaried workers’ compensated diseases dataset, which is also available at the workplace level. Comparisons with rnv3p data will allow a more systemic picture of OD across territories to be drawn, and to reassess on-site prevention measures since this information is available on a workplace scale. Data could also be compared with indicators from the Uncompensated Work-Related Diseases Network [22], which draws estimates of non-compensated work-related illnesses at the regional level. Finally, complementary approaches centred on different types of exposures are also needed to advance risk-assessment.

Our work calls for further action. For instance, it would be of interest to examine employment area which have shown no case capture to date (shaded zones of the network). This can be done by initiating exchanges between the nearest OD Clinic and the occupational health services covering these areas.

In the literature, we found no other studies in the occupational health field that attempted to describe this kind of network from a geographical point of view. We also found no other studies which analysed GIS data pertaining to Occupational Diseases, except those cited above for which the limitations are already mentioned.

In France, a GIS-based project aimed at collecting statistical data (mainly about employment) in order to map “work territories” (the ATTLAS project) was initiated in 2006. This project built an open-access on-line tool allowing several indicators to be mapped at different scales [23]. Another research program (GISCOP 93), is using geographic tools such as GIS in order to collect and map risks of occupational exposure to carcinogens in a particular French département located in the Ile de France Region [24]. Unfortunately, neither of these projects includes data regarding work-related health.

This work, as well as those mentioned above that also use a geographic approach, is in line with one major objective of the French National Plan for Occupational Health 2016–2020, related to knowledge building [25]. Especially the “action 3.13” objective is to give all regions possibilities to identify and map geographically relevant information pertaining to employment and occupational health, in order to build territorial diagnoses of occupational health. The ultimate goal is to promote a regional approach to occupational health risk assessment, prioritize actions, and facilitate cooperation between all players concerned by OH, so as to improve prevention.

## Conclusion

The geographic approach to this network, enhanced by the possibilities provided by the GIS tool, allow a better understanding of the coverage of this network at a national level, as well as the visualization of capture rates for all OD clinics. Highlighting geographic and thematic shading zones bring new perspectives to the analysis of occupational health data, and should improve occupational health vigilance and surveillance.

## Additional files

10.1186/s12942-016-0063-7Employment area with higher catchment of OD by the French OD clinics network (rnv3p) for the broad categories of occupational activities (namely: A. agriculture, B. construction, C. commercial services and D. non-commercial services). Legend. Occupational diseases (OD) reported to the rnv3p by OD Clinics are reported to the expected number for each employment area. For each of these geographical units, the sum of the ratios of observed vs expected OD for each category of activity was calculated and mapped. Sources: rnv3p 2001–2012, and French National Institute of Statistics and Economic Studies (INSEE). For industry, see Fig. 6.
10.1186/s12942-016-0063-7Success in geocoding rnv3p observations, according to the latency of diseases, and for each French OD clinic (rnv3p 2001–2012).

## Abbreviations

EA: employment area (geographic entity level for which economic activities figures are given by INSEE; In French “zones d’emplois”)
GIS: Geographic Information Systems
ICD-10: International Classification of Diseases (10th version)
INSEE: French National Institute of Statistics and Economic Studies (“Institut National de la Statistique et des Etudes Economiques”)
NAF: national activity codes (“Nomenclature d’Activités Française”)
OD: occupational diseases
PNSM: French National Mesothelioma Surveillance Program (“Programme National de Surveillance du Mésothéliome”)
rnv3p: French National Occupational Diseases Surveillance and Prevention Network (“Réseau National de Surveillance et de Prévention des Pathologies Professionnelles”)
SDE: standard deviation ellipses
WRD: work-related diseases
