# Supplementary figures and images for: Geographic dimensions of a health network dedicated to occupational and work related diseases
Source: Int J Health Geogr. 2016 Sep 27;15:34. doi: 10.1186/s12942-016-0063-7 (PMC5039888; doi:10.1186/s12942-016-0063-7)

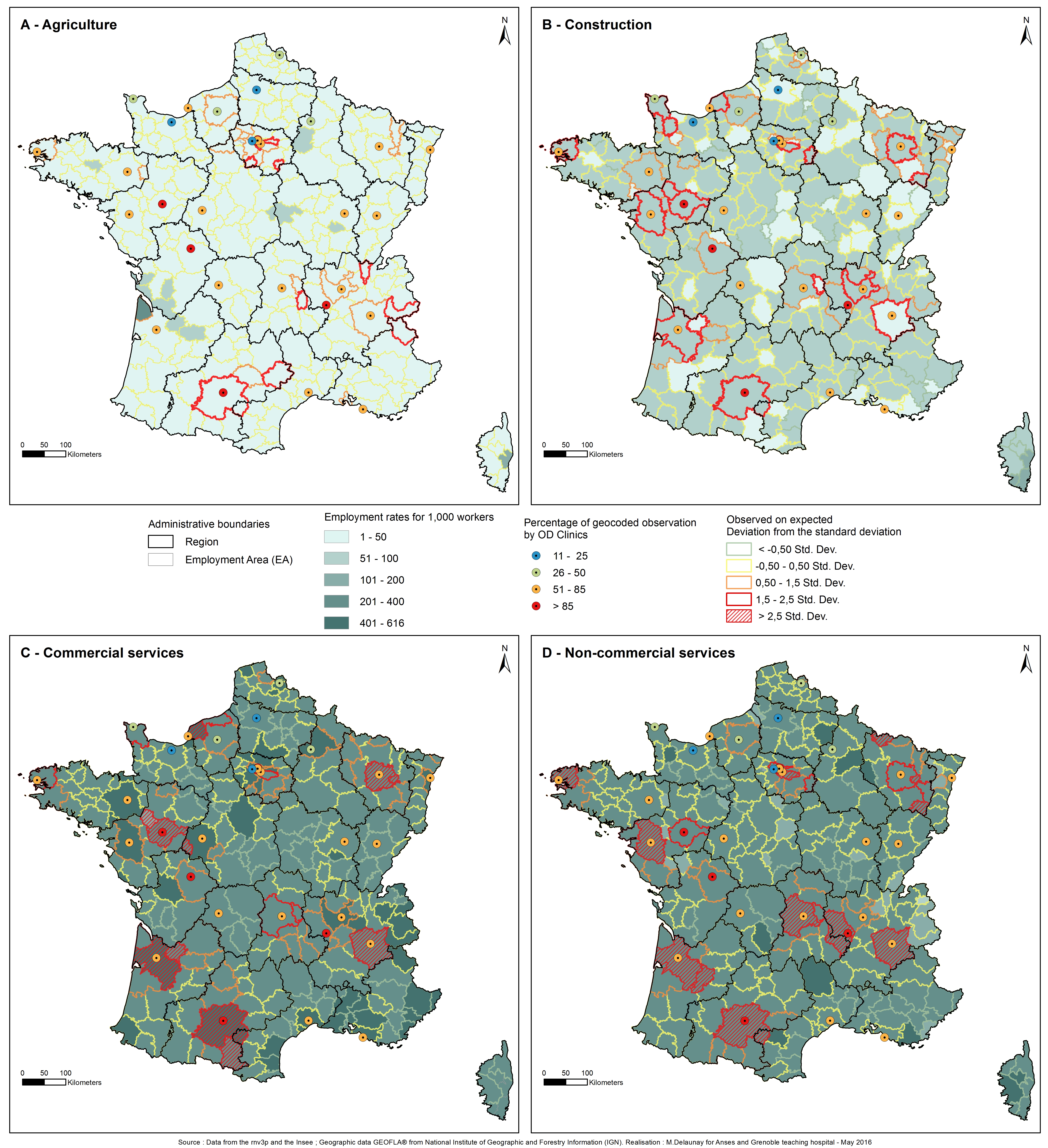

Supplement: Supplementary file 1 — 10.1186/s12942-016-0063-7Employment area with higher catchment of OD by the French OD clinics network (rnv3p) for the broad categories of occupational activities (namely: A. agriculture, B. construction, C. commercial services and D. non-commercial services). Legend. Occupational diseases (OD) reported to the rnv3p by OD Clinics are reported to the expected number for each employment area. For each of these geographical units, the sum of the ratios of observed vs expected OD for each category of activity was calculated and mapped. Sources: rnv3p 2001–2012, and French National Institute of Statistics and Economic Studies (INSEE). For industry, see Fig. 6. [file 12942_2016_63_MOESM1_ESM.jpg]
