# Supplementary material for: Geographic dimensions of a health network dedicated to occupational and work related diseases
Source: Int J Health Geogr. 2016 Sep 27;15:34. doi: 10.1186/s12942-016-0063-7 (PMC5039888; doi:10.1186/s12942-016-0063-7)
Supplement: Supplementary file 2 — 10.1186/s12942-016-0063-7Success in geocoding rnv3p observations, according to the latency of diseases, and for each French OD clinic (rnv3p 2001–2012). [file 12942_2016_63_MOESM2_ESM.docx]

**Table S1.** Success in geocoding rnv3p observations, according to the latency of diseases, and for each French OD clinic (rnv3p 2001-2012).

|  | **Long latency pathologies**  Cancers (C*), pneumoconioses (J92*, J60-J64*) | | | **Other pathologies**  Rhinitis (J30*, J31*), asthma (J45*), contact dermatitis (L23*, L24*, L25*) | | |
| --- | --- | --- | --- | --- | --- | --- |
| **OD Clinics** | Total of observations | Linked to the workplace | Geolocated | Total of observations | Linked to the workplace | Geolocated |
| Amiens | 262 | 43 | 30 (11%) | 62 | 37 | 29 (47%) |
| Angers | 143 | 101 | 95 (66%) | 48 | 47 | 47 (98%) |
| Besançon | 93 | 47 | 36 (39%) | 64 | 53 | 45 (70%) |
| Bordeaux | 2,883 | 808 | 674 (23%) | 879 | 817 | 699 (80%) |
| Brest | 2,449 | 631 | 614 (25%) | 560 | 476 | 467 (83%) |
| Caen | 2,424 | 166 | 120 (5%) | 434 | 124 | 99 (23%) |
| Cherbourg | 616 | 105 | 66 (11%) | 44 | 28 | 23 (52%) |
| Clermont | 412 | 162 | 145 (35%) | 714 | 745 | 687 (96%) |
| Cochin | 680 | 286 | 273 (40%) | 3,315 | 3,168 | 3,135 (95%) |
| Créteil | 3,179 | 754 | 714 (22%) | 2,140 | 1,867 | 1,835 (86%) |
| Dijon | 47 | 34 | 32 (68%) | 69 | 69 | 65 (94%) |
| Fernand Widal | 268 | 234 | 224 (84%) | 1,377 | 1,302 | 1,278 (93%) |
| Garches | 678 | 93 | 82 (12%) | 1,317 | 413 | 360 (27%) |
| Grenoble | 2,293 | 379 | 368 (16%) | 1,190 | 1,150 | 1,052 (88%) |
| Hôtel-Dieu | --- | --- | --- | --- | --- | --- |
| Le Havre | 2,147 | 932 | 882 (41%) | 134 | 101 | 82 (61%) |
| Lille | 3,329 | 781 | 554 (17%) | 645 | 538 | 394 (61%) |
| Limoges | 127 | 79 | 69 (54%) | 20 | 18 | 18 (90%) |
| Lyon | 1,729 | 395 | 369 (21%) | 1,304 | 925 | 882 (68%) |
| Marseille | 471 | 271 | 247 (52%) | 741 | 689 | 638 (86%) |
| Montpellier | 192 | 49 | 41 (21% | 221 | 174 | 159 (72%) |
| Nancy | 1,070 | 829 | 292 (27%) | 464 | 432 | 399 (86%) |
| Nantes | 538 | 233 | 142 (26%) | 2,504 | 2,499 | 1,432 (57%) |
| Poitiers | 60 | 48 | 46 (77%) | 74 | 70 | 70 (95%) |
| Reims | 247 | 77 | 72 (29%) | 453 | 333 | 319 (70%) |
| Rennes | 64 | 41 | 41 (64%) | 126 | 118 | 116 (92%) |
| Rouen | 1,379 | 289 | 262 (19%) | 346 | 277 | 257 (74%) |
| Saint-Etienne | 78 | 38 | 34 (44%) | 186 | 182 | 172 (92%) |
| Strasbourg | 508 | 192 | 177 (35%) | 192 | 165 | 158 (82%) |
| Toulouse | 196 | 116 | 116 (59%) | 1,078 | 1,061 | 1,049 (97%) |
| Tours | 553 | 230 | 213 (39%) | 232 | 200 | 186 (80%) |
| **TOTAL** | **29,115** | **8,443** | **7,030 (24%)** | **20,933** | **18,078** | **16,152 (77%)** |

Legend. The diseases codes refer to the ICD-10 classification
